# Observation of spin-polarized Anderson state around charge neutral point in graphene with Fe-clusters

**DOI:** 10.1038/s41598-020-61481-6

**Published:** 2020-03-16

**Authors:** Jungmin Park, Inseon Oh, Mi-Jin Jin, Junhyeon Jo, Daeseong Choe, Hyung Duk Yun, Suk Woo Lee, Zonghoon Lee, Soon-Yong Kwon, Hosub Jin, Suk Bum Chung, Jung-Woo Yoo

**Affiliations:** 1 School of Materials Science and Engineering, Ulsan National Institute of Science and Technology, Ulsan, 44919 Republic of Korea; 2 Center for Scientific Instrumentation, Division of Scientific Instrumentation & Management, Korea Basic Science Institute, Daejeon, 34133 Korea; 3 Center for Multidimensional Carbon Materials, Institute for Basic Science (IBS), Ulsan, 44919 Republic of Korea; 4 Department of Physics, Ulsan National Institute of Science and Technology, Ulsan, 44919 Korea; 5 Department of Physics, University of Seoul, Seoul, 02504 Korea

**Keywords:** Surfaces, interfaces and thin films, Graphene

## Abstract

The pristine graphene described with massless Dirac fermion could bear topological insulator state and ferromagnetism via the band structure engineering with various adatoms and proximity effects from heterostructures. In particular, topological Anderson insulator state was theoretically predicted in tight-binding honeycomb lattice with Anderson disorder term. Here, we introduced physi-absorbed Fe-clusters/adatoms on graphene to impose exchange interaction and random lattice disorder, and we observed Anderson insulator state accompanying with Kondo effect and field-induced conducting state upon applying the magnetic field at around a charge neutral point. Furthermore, the emergence of the double peak of resistivity at *ν* = 0 state indicates spin-splitted edge state with high effective exchange field (>70 T). These phenomena suggest the appearance of topological Anderson insulator state triggered by the induced exchange field and disorder.

## Introduction

Two-dimensional(2D) topological insulator has helical edge boundary due to spin-orbit coupling (SOC) and time reversal symmetry leading to quantum spin Hall (QSH) phase^1^. This nontrivial topological phase could be also emerged when disorder is added to a trivial band structure^2–7^. This disorder-driven topological state, *i.e*. topological Anderson insulator (TAI), was first predicted in metallic 2D quantum wall^2,3^. The theoretical studies have shown that the TAI phenomena could be generic for disordered systems and both topology and disorder have rich combined influences on the quantum transport^2–5^. Yet, its experimental demonstration was recently achieved only in precisely controlled optical lattice^6,7^.

Electron band structure of pristine graphene has been extensively studied over the last decade because of its unusual transport properties^8,9^, and it has been shown that physical properties of graphene can be strongly modified when it is functionalized with various adatoms^10–23^, or proximity effect from heterostructures^24–30^. Disordered graphene by heavy adatoms could exhibit diverse condensed matter phenomena such as spin Hall effect and QSH state (topological insulator state) due to the instilled spin-orbit coupling and honeycomb lattice distortion. For example, chemically or physically decorated adatom in graphene could exhibit spin-charge conversion known as spin Hall effect due to induced strong spin-orbit coupling^21–23^. Also, it was theoretically predicted that dilute heavy adatoms, such as platinum, indium and thallium, could lead to a robust QSH state in graphene, with a band gap exceeding that of pure graphene by many orders of magnitude^14^. Even the pristine graphene was predicted to exhibit a QSH phase and have a nontrivial topological order with an energy gap generated by the intrinsic spin-orbit coupling at ultra-low temperature^31^.

When graphene is laminated on the ferromagnetic insulator, it could acquire local moment and/or experience magnetic exchange field^27–30^, as evidenced through the Zeeman Hall effect^28,29^ or the anomalous Hall^27^ effect in graphene/magnetic insulator heterostructures. In particular, *p*-wave nature of hybridization between conduction electrons and localized state makes induced local moment highly stable^32^. The intense exchange field lifts the ground-state degeneracy of graphene in the quantum Hall state, leading to spin-polarized *v* (filling factor of Landau level) = 0 state similar to the quantum spin Hall state or the quantum Hall metal state^28–30^. Exchange field and spin polarized density of state can be also induced by magnetic adatoms, such as Co and Fe^15,33^. Moreover, random distribution of magnetic adatoms accompanies disorder leading to Anderson localization^34^ in the vicinity of the Dirac point^18^. Here, the presence of the exchange coupling between the itinerant and local spins can further enhance Anderson localization above Kondo temperature^18^. Anderson metal insulator transition (MIT) upon changing carrier density was also predicted when adatoms is on the center of the honeycomb hexagon forming impurity plaquette^11,16^.

In this study, we employed magnetic impurities of Fe clusters/adatoms on graphene to induce strain and exchange field simultaneously. Results showed that the magnetic field induced conducting state, which suggests the emergence of spin-polarized Anderson state in the graphene. Near the charge neutral point (CNP) of graphene, the Anderson insulator state appeared in company with Kondo effect and it can be transited into a metallic state with a spin-splitted edge state under the high magnetic field, similar to a TAI state.

## Results and Discussions

**Figure 1:**
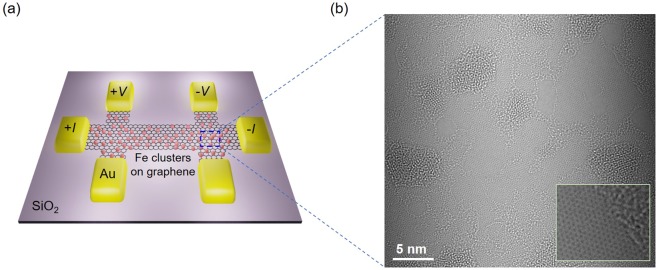
Graphene with Fe clusters. (**a**) Schematic illustration of H-bar graphene device with Fe clusters. (**b**) TEM image of single layer graphene with Fe clusters. Random distribution of Fe clusters was made by 0.5 nm e-beam deposition. The inset displays enlarged view of Fe cluster edge on graphene.

Figure 1(a) illustrates studied graphene Hall-bar device with Fe clusters (see Materials and Methods for details). The geometry of Hall-bar device has a channel width (*w*) of ~ 2 *μ*m and lengths (*L*_s_) of ~ 6 *μ*m. Fe was physically deposited by e-beam evaporation (~0.5 nm), which forms random distribution of clusters/adatoms on the surface of graphene. Then 20 nm of Al_2_O_3_ was deposited for capping layer. Random distribution of Fe clusters can be clearly observed in transmission electron microscopy image shown in Fig. 1(b). Edge of Fe clusters on graphene is displayed in the inset of Fig. 1(b). Raman spectra of Fe-clusters graphene displays shift and broadening of 2D peak suggesting that Fe clusters and Al_2_O_3_ capping layer induced significant strain in underlying graphene^35^ (see Supplementary Information (SI) Fig. S1). It was theoretically reported^15^ that the adsorption of the adatoms on graphene can generate significant in-plane and vertical distortion in lattice of graphene, And this random strain by the adatoms can give rise to Anderson localization in the vicinity of the CNP^11^.

**Figure 2:**
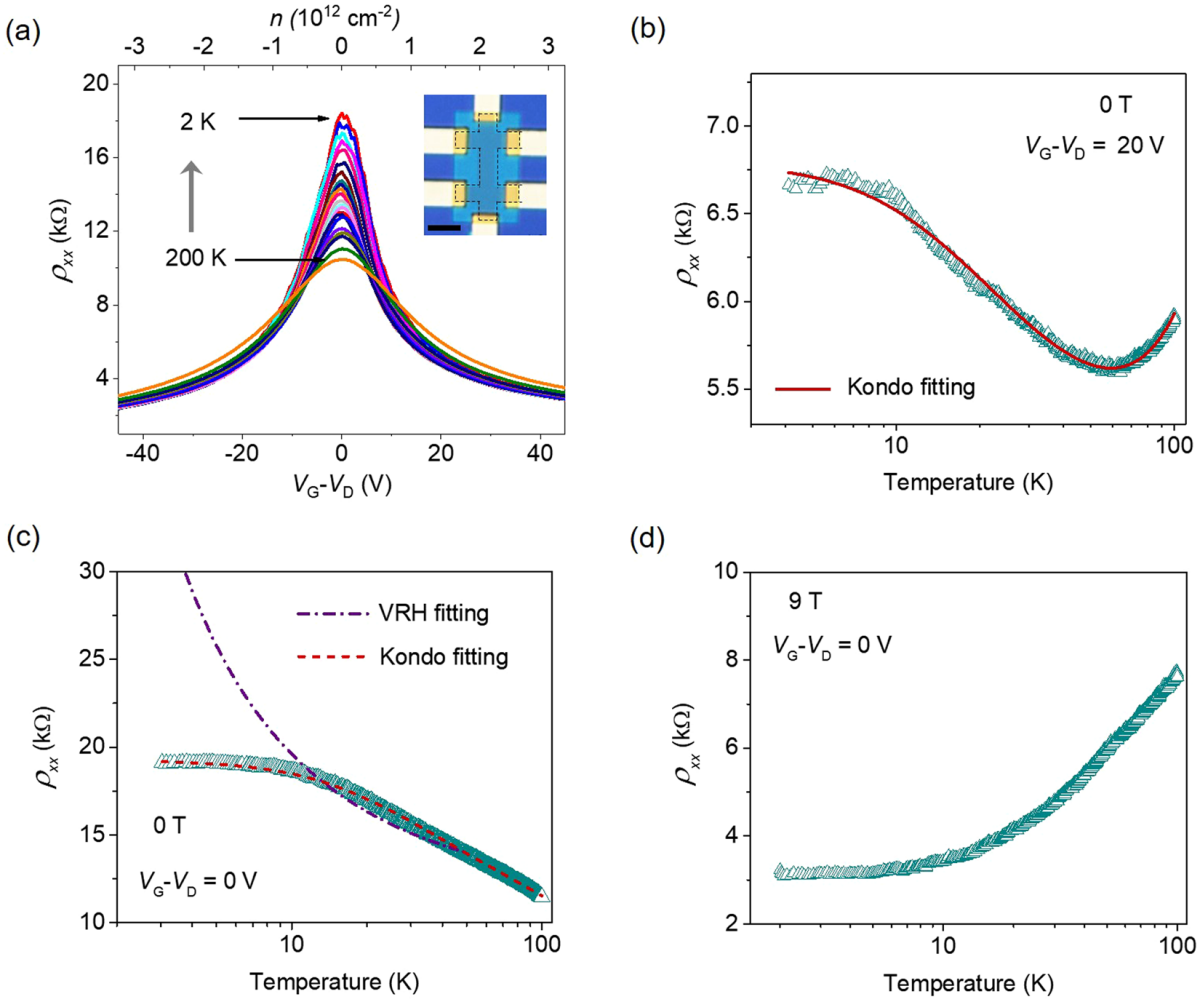
Characteristics of charge transport in graphene with Fe clusters. (**a**) Resistivity as a function of gate voltage (carrier density *n*) measured at various temperature. Inset exhibits optical image of the fabricated device. Scale bar is 5 *μ*m. Temperature dependent resistivity measured at *V*_G_ − *V*_D_ = +20 V (**b**) and at CNP (**c**) in the absence of magnetic field. Red solid line is a fitting curve with Eq. (1). Red dashed line indicates *R*_K_(*T*/*T*_K_) of Kondo model. Purple dash-dotted line shows variable-range hopping model. (**d**) Temperature dependent resistivity at CNP in the presence of the applied magnetic field 9 T, indicating metallic state.

Figure 2(a) displays gate dependent *ρ*_*xx*_ upon varying temperature. The estimated mobility, *μ* = 1/*e* × *dσ/dn*, is about 600 cm^2^/V∙s at 2 K. This value of mobility is significantly lower than that of the pristine graphene, which can be attributed to the strain induced disorder by Fe particle plaquette as shown in Raman spectra (see Supplementary Information (SI) Fig. S1). The temperature dependent *ρ*_*xx*_ exhibits two different regimes. At around CNP, *ρ*_*xx*_(*T*) shows insulating behavior, while it exhibits metallic behavior at high carrier density. The resistance of graphene on SiO_2_/Si substrate generally decreases with decreasing temperature in accordance with its semi-metallic band structure^36^. But it displays insulating behavior at around CNP in high magnetic field due to quantum phase transition induced by strong localization of electrons^37^. The MIT upon varying carrier density has been also observed on ultraclean graphene having high mobility (~200,000 cm^2^/V∙s) in the absence of magnetic field (e.g. suspended graphene^38^, graphene on h-BN^39^, etc). In suspended graphene, the insulator state near CNP is a result of a strongly reduced charge inhomogeneity^38^. In the case of graphene on h-BN, broken valley symmetry can give rise to insulating behavior^39^. However, disordered graphene such as hydrogenated graphene and graphene exposed to ozone, only exhibits insulating state regardless of carrier density and external magnetic field due to bandgap opening^13,17^. In our study, although Fe-clusters graphene device exhibits low mobility and diffusive transport by disorder, the MIT by changing gate voltage appear in the absence of magnetic field, similar to the case of ultraclean graphene^38,39^ or to the case of graphene on SiO_2_ under high magnetic field^37^. In disordered graphene, this MIT by changing gate voltage can be attributed to the presence of mobility edge which separates Anderson localized state near CNP from metallic state at high carrier density^11^.

Figure 2(b) displays low temperature variation of resistivity in the metallic regime. Result shows strong upturn in *ρ*_*xx*_ below 50 K. And *ρ*_*xx*_ was nearly saturated with decreasing temperature further. This behavior could be attributed to the Kondo effect due to the strong correlation between itinerant electrons and magnetic impurity. The Kondo effect on graphene due to magnetic adatoms^12,40–42^ and/or defect-induced moments^43,44^ was theoretically predicted. The temperature dependent resistivity of the conventional Kondo model can be described as follows^45,46^.

1 $$\begin{array}{ccc}{R}_{{\rm{kon}}}(T) & = & {R}_{0}+q{T}^{2}+p{T}^{5}+{R}_{{\rm{K}}}(\frac{T}{{T}_{{\rm{K}}}})\\ {R}_{{\rm{K}}}\left(\frac{T}{{T}_{{\rm{K}}}}\right) & = & {R}_{{\rm{K}}}(T=0){\left(\frac{{T}_{{\rm{K}}}^{\text{'}2}}{{T}^{2}-{T}_{{\rm{K}}}^{\text{'}2}}\right)}^{S}\end{array}$$

where *R*_0_ represents the resistance from sample disorder. *T*^2^ and *T*^5^ terms are the electron-electron and electro-phonon interaction, respectively. *T*_K_ indicates Kondo temperature. The *R*_K_(*T*/*T*_K_) is a function representing the universal behavior of Kondo effect, *i.e*. logarithmic increase of resistivity below *T*_K_ and its saturation at very low *T*. Here, ${{T}^{{\rm{{\prime} }}}}_{{\rm{K}}}={T}_{K}/({2}^{1/s}-1{)}^{1/2}$ and *s* = 0.22 ± 0.01 obtained by renormalization group^46^. We fixed *s* = 0.21 for fitting. The overall behavior of *ρ*_*xx*_(*T*) at *V*_G_ − *V*_D_ = +20 V can be well fitted to the Eq. (1) as shown in Fig. 2(b). The *ρ*_*xx*_ (*T*) at higher carrier density also follows well to the universal behavior of Kondo effect (see Supplementary Information (SI) Fig. S2). Figure 2(c) displays measured *ρ*_*xx*_(*T*) at CNP, where overall *T* dependence shows insulating behavior. For the disorder graphene showing insulating behavior, the temperature dependence of resistance can be described by either variable-range hopping (VRH)^13,17^ or the universal function of the Kondo model^47^. The VRH in two-dimension has a characteristic temperature dependence of $\,\rho =\,{\rho }_{o}{e}^{{({T}_{0}/T)}^{1/3}}$, indicating divergence of resistance at low temperature. As shown in Fig. 2(c), the observed low temperature dependence of resistance at CNP is saturated and well fitted with simple Kondo model than VRH. Here, the *T*^2^ and *T*^5^ terms are nearly negligible at CNP while *R*_0_ shows peak at CNP^47^. The Kondo temperature at CNP and *V*_G_ − *V*_D_ = +20 V is 95 K and 69 K, respectively. The estimated Kondo temperatures are in order of several 10 K (see Supplementary Information (SI) Fig. S2 for Kondo temperature with various gate voltage). These values are in general much higher than that of s-wave Kondo system because hybridization of conduction electrons with localized states in graphene lead to *p*-wave hybridization, which typically results in higher Kondo temperature as discussed in S. A. Jafari *et al*.^42^. In addition, excitation of spin-1 boson due to inter-band particle-hole processes in graphene could further enhance the Kondo effect^48,49^.

Figure 2(d) displays temperature dependent *ρ*_*xx*_ at CNP in the presence of the high magnetic field 9 T. Results shows that the applied high magnetic field convert the system into conducting state at CNP. This behavior is also in agreement with Anderson localized state at CNP predicted in ref. ^11^. The observed field-induced conducting state can be observed only in the presence of Fe clusters/adatoms on graphene, (see Supplementary Information (SI) Fig. S3), which we will discuss later in detail.

**Figure 3:**
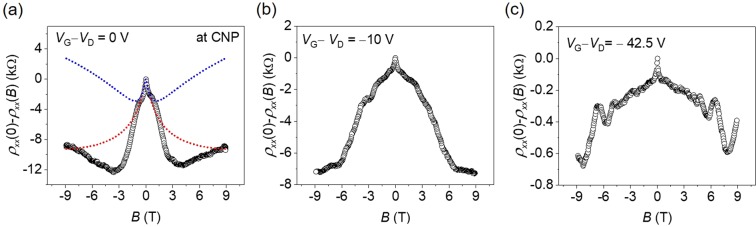
Magnetoresistance upon applying perpendicular magnetic field at 2 K. Magnetoresistance curve measured at CNP (**a**), *V*_G_ − *V*_D_ = −10 V (**b**), and *V*_G_ − *V*_D_ = −42.5 V (**c**). The dotted line indicates fitting curve from Eq. (2). The dotted blue line shows the weak localization fitting for the low-field magnetoresistance behavior. The dotted red line is a fitting curve by increasing valley scattering without changing phase coherence time.

We then performed the magnetoresistance measurement to investigate localization behavior further. Figure 3 shows measured magnetoresistance upon applying perpendicular magnetic field at various gate voltage. In all cases, the magnetoresistances were negative, and the Shubnikov-de Hass oscillations were observed at high carrier density. To find the influence of quantum interference near the CNP, we fit our data according to a localization theory developed for graphene^50^, where the correction to the semiclassical resistivity is given by

2 $$\begin{array}{ccc}\Delta \rho (B) & = & -\frac{{e}^{2}{\rho }^{2}}{\pi h}\left[F\,\left(\frac{B}{{B}_{\varphi }}\right)-F\,\left(\frac{B}{{B}_{\varphi }+2{B}_{{\rm{inter}}}}\right)-2F\,\left(\frac{B}{{B}_{\varphi }+{B}_{{\rm{intra}}}}\right)\right]\\ F({\rm{z}}) & = & \mathrm{ln}\,z+\psi \left(\frac{1}{2}+\frac{1}{z}\right)\,{B}_{\varphi ,{\rm{inter}},{\rm{intra}}}=\frac{\hslash }{4De}{\tau }_{\varphi ,{\rm{inter}},{\rm{intra}}}^{-1}\,\end{array}$$

where *ψ* is digamma function, *D* is the diffusion coefficient, ${\tau }_{{\rm{inter}}}^{-1}$ is intervalley scattering rate, ${\tau }_{{\rm{intra}}}^{-1}$ is intravalley scattering rate, and ${\tau }_{\varphi }^{-1}$ is dephasing rate. The fit to Eq. (2) is shown in Fig. 3(a). Here, we plot $\Delta \rho =\rho (0)-\rho (B)$ from experimental data. The dotted blue line is fitting curve for low-field magnetoresistance of weak localization behavior (${\tau }_{\varphi }=0.3\,{\rm{ps}},\,{\tau }_{{\rm{inter}}}\,=\,0.14\,{\rm{ps}},\,{\tau }_{{\rm{intra}}}=\,0.05\,{\rm{ps}}$). The suppression of resistivity at intermediate field can be obtained by increasing both inter- and intra- valley scattering times (${\tau }_{\varphi }=\,0.3\,{\rm{ps}},\,{\tau }_{{\rm{inter}}}\,=\,0.01\,{\rm{ps}},\,{\tau }_{{\rm{intra}}}=\,0.014\,{\rm{ps}})\,\,$without changing phase coherence time (${\tau }_{\varphi })$ (dotted red line in the Fig. 3(a). Here, ${\tau }_{\varphi }$ mainly affect low-field magnetoresistance and nearly insensitive to the magnetoresistance at higher field. Note, that this upper limit of phase coherence time is much shorter than that estimated in pristine graphene (${\tau }_{\varphi } \sim 10\,{\rm{ps}})$^51^, which is possibly due to strong magnetic dephasing mechanism as discussed in Lundeberg *et al*.^52^. The fitting to low field region allows us to extract a value for phase coherence length ${L}_{\varphi }=\sqrt{{\tau }_{\varphi }D}$ with the diffusion coefficient (*D*) ~ 0.01. The obtained phase coherence length at around CNP was ~ 57 nm. The localization length is given by^13,17^

3 $${\xi }_{D}\cong {L}_{e}exp\left(\frac{{\sigma }_{D}}{{e}^{2}/h}\right)$$

where, *L*_e_ is the elastic length from *L*_e_ = ${\sigma }_{D}h/2{e}^{2}{(\pi n)}^{1/2}$, *σ*_*D*_ is Drude conductivity, and *n* is charge carrier density. The estimated localization length at around CNP is ~3.2 nm (~160 nm at *V*_G_ − *V*_D_ = −10 V and ~50 *μ*m at *V*_G_ − *V*_D_ = −42.5 V). The estimated localization length at CNP in pristine graphene is typically in order of 100 nm^53^. The observed short localization in our studied system can be attributed to the presence of random strain induced from Fe clusters/adatoms. Because the phase coherence length is much larger than localization length, this indicates the system is in the strong localization (called Anderson localization) regime. Based on the localization length scale near CNP, the Fermi velocity was estimated to be ~2 × 10^5^ m/s, whose energy is in order of ~100 meV. This energy scale in the vicinity of CNP corresponds to the mobility edge reported in previous theoretical study^11^. Note that the strong suppression in magnetoresistance under high magnetic-field in Fig. 3a,b reflects that the system is in Quantum Hall regime.

The original description of Kondo effect is closely related to Anderson impurity model. A low energy features of Anderson Hamiltonian is equivalent to those of Kondo Hamiltonian with exchange interaction^54–56^. As previously mentioned, it was theoretically predicted^18^ that magnetic adatoms (Co, Fe) on graphene can enhance Anderson localization with spin-polarized density of state in the vicinity of Dirac point. Thus, our observation of both Kondo effect and Anderson localization is consistent with previous theoretical studies.

**Figure 4:**
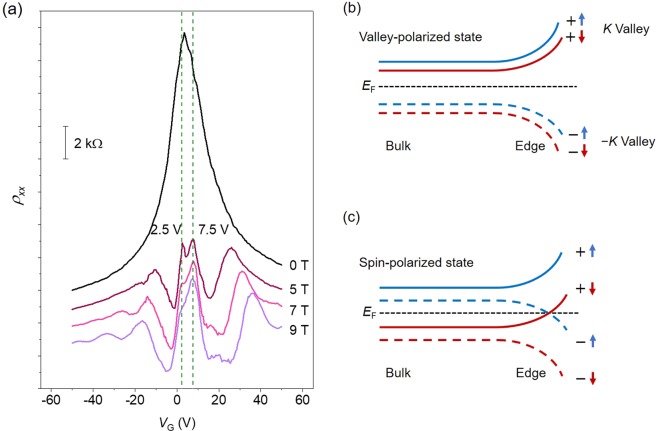
The splitting of zeroth Landau level in Fe- clusters graphene device. (**a**) The resistivity as a function of gate voltage measured at 2 K with various applied magnetic field of 0 T, 5 T, 7 T, and 9 T. The splitting at around CNP was observed and the resistance peak was shifted from 3.5 V to 7.5 V with increases magnetic field. The *ρ*_*xx*_ (*V*_G_) curves are shifted vertically for clarity. (**b**) Schematic illustration of valley polarized state with bulk gap. In this quantum insulator state, the spin splitting is weak. (**c**) Schematic illustration of spin-polarized state by Zeeman splitting. A counter-propagating edge state with opposite spin polarization exist at around Dirac point similar to quantum spin Hall effect. Blue line (red line) indicates spin-up state (spin-down state). Solid line and + (dashed lines and −) represent *k* valley (−*k* valley).

The solution of Anderson Hamiltonian gives the mobility edges that separate localized and extended state. Anderson insulator state appears when a fermi energy lies in the localized state. Thus, transition into metallic state under high magnetic field as shown in Fig. 2(d) reflects that a conducting channel is developed due to either delocalization^57^ or edge state^28^. In order to investigate further about the conducting state under high magnetic field, we measured gate-dependent resistivity with varying applied magnetic field. As shown in Fig. 4(a), the CNP was initially located at 3.5 V in the absence of magnetic field. With increasing external magnetic field, the resistivity at around CNP develops double-peak features (at 7.5 V and 2.5 V), as shown in Fig. 4(a). Besides, further increase of magnetic field reduced the magnitude of peak at 2.5 V. Finally, the peak observed at 2.5 V was strongly suppressed at 9 T. In short, the CNP shift from 3.5 V to 7.5 V upon increasing applied magnetic field. The shift of CNP can be also clearly observed in the plot of conductance *G* vs *V*_g_ (see Supplementary Information (SI) Fig. S4), which exhibits base conductance of 2(*e*^2^*/h*).

Previous theoretical and experimental reports provided that the *ν* = 0 state could be a spin-polarized state with gapless chiral edge mode, a valley-polarized state without gapless chiral edge mode or the intermediate state between spin and valley polarized^28,30,58^. The valley-polarized state should lead to increasing longitudinal resistivity with increasing magnetic field, while, in the case of spin-polarized state, metallic behavior appear due to edge state^28^, as illustrated in Fig. 4b,c. Here, the strong interfacial magnetic exchange field can generate the spin-polarized edge transport at *ν* = 0 state originating from Zeeman splitting, and this edge transport will appear as the double peak of longitudinal resistivity near the Dirac point indicating the presence of counter-propagating edge channels like the quantum spin Hall effect^28,30^. In our sample, we observed that the double peak of longitudinal resistivity near CNP as shown Fig. 4(a) and that the resistivity with increasing magnetic field was reduced, indicating spin-polarized edge transport at *ν* = 0 state. For the applied magnetic field of 9 T, the magnetic length of graphene is about $\,26/\sqrt{B}$ nm ~ 8.8 nm. The estimated localization length is ~3.2 nm at around CNP. In general, the magnetic length should be shorter than localization length to induce phase transition (from Anderson insulator to quantum metallic state)^57,59^. In this study, although localization length is shorter than magnetic length, the quantum metallic state with spin-polarized edge exists at around CNP. The graphene π-orbitals can have strong hybridization with the Fe 3*d* orbitals, and the first-principle calculation predicted a proximity-induced exchange field up to ~ 1100 T^60^. Experimentally, it was shown that the graphene on BiFeO_3_ substrate has effective exchange field about 280 T^29^. In our study, effective exchange field should be higher than 70 T to generate quantum phase transition as shown in Figs. 2(d) and 4(a). In addition, Wu *et al*., predicted^61^ the shift of Dirac point with spin-polarization for strained graphene with effective exchange field by using low-energy effective Hamiltonian. It was also reported that the combined play of exchange field and spin-orbit interaction could cause asymmetric spin splitting of Dirac state in the graphene^20^. So, we attribute double-peak and shift of CNP shown in Fig. 4 to the effective exchange field and the strain induced by Fe- clusters/adatoms.

Recently, it has been theoretically proposed that the topological Anderson insulator state is a more universal phenomenon and can appear in Kane-Mele model on a honeycomb lattice with Anderson disorder contribution^2,4^. The primary difference from conventional topological insulator is that Fermi energy lies within mobility gap in place of real band gap. The gapless edge states are between two extended state (mobility gap). In the strong disorder limit, topological Anderson state will eventually disappear and become trivial band structure because all states are localized without extended state^62^.

Physical absorption of other magnetic adatoms could also induce similar effect. Xiaojie Liu *et al*. predicted^15^ the reduced magnetic moments for the magnetic adatoms (Fe, Co, Ni) on graphene and compared magnetic moments of the corresponding isolated atoms. According to this study, the net magnetic moments for Fe, Co, Ni can be reduced from 4.0, 3.0, and 2.0 *μ*_B_ (for isolated atoms) to 2.0, 1.0, 0 *μ*_B_ in adatoms/graphene systems, respectively, due to electrons transfer. Thus, the Kondo effect or spin polarized state in Ni/graphene system could not be observed as the net magnetic moment in Ni/graphene system is zero.

## Conclusions

We showed that magnetic impurities of Fe clusters/adatoms on graphene can effectively induce strain and exchange field simultaneously. The induced strain and exchange field (>70 T) by Fe particles lead to Anderson localization with Kondo effect at around CNP. In addition to Anderson localization, spin polarized edge state and the shift of CNP were emerged by applying a high magnetic field. These results provide importance insights for spin-polarized Anderson transition in two-dimensional honeycomb lattice. Our study showed that graphene with random distribution of magnetic clusters/adatoms is a good test-bed for the investigation of TAI.

## Materials and Methods

### Synthesis and transfer of graphene

A monolayer graphene was grown on a polycrystalline Cu foil using a chemical vapor deposition method demonstrated elsewhere^23^. 25 *μ*m copper foil (Alfa Aesar, 99.8% purity) was electropolished in phosphoric acid for 15 min and rinsed with distilled water followed by isopropyl alcohol (IPA). The copper foil was loaded into a quartz tube 3-zone furnace and the temperature was increased to 1050 °C in the H_2_ environment for removal of native oxides in the copper with surface reconstruction. Monolayer graphene was synthesized by introducing CH_4_ gas under H_2_ gas insertion with a ratio of 10:5 (sccm) for 15 min, and transferred onto the Si/SiO_2_ (300 nm) subatrate using a polymethyl methacrylate (PMMA) wet transfer process. To remove possible resist residues, samples were annealed in low vacuum at 300 °C.

### Fabrication of Fe-clusters/adatoms graphene Hall bar device

The pattern for Hall bar and Au electrode was fabricated by electron beam lithography. Hall bar geometry of graphene was defined via oxygen plasma etching. Thermally deposited Au (60 nm)/ Cr (3 nm) was used for electrode. Ultrathin Fe layer (~0.5 nm) was deposited by e-beam evaporation with a deposition rate of 0.05 Å/s. Finally, 20 nm of Al_2_O_3_ layer was deposited via e-beam evaporation to protect from unwanted oxidation and contamination.

### Characterization of Fe-clusters/adatoms graphene

The Raman spectroscopy was performed by Alpha 300 R spectrometer (WITec) with a 532 nm laser source. The spot size of laser source was ~ 1 *μ*m in diameter and the laser power was ~1 mW. High resolution transmission electron microscopy (HRTEM) images were acquired using an aberration-corrected Titan cube G2 operated at 80 kV

### Electrical measurement

Electrical measurements were performed in a Quantum Design Physical Property Measurement System (PPMS) with the Keithley source meter (K2636) and a nano-voltmeter (K2182). The indium (In) with copper wire was used for the electrical contacts to Au pads of the device. The samples are loaded to a vacuum chamber of PPMS which can control a variable temperature (2 K~300 K) and magnetic field (−9 T~+9 T). The sample was annealed in vacuum chamber of PPMS at about 100 °C to remove moisture on graphene before electrical measurement. The 500 nA (dc) was applied for electrical transport.

## Supplementary information

Supplementary information.
