# Supplementary material for: Observation of spin-polarized Anderson state around charge neutral point in graphene with Fe-clusters
Source: Sci Rep. 2020 Mar 16;10:4784. doi: 10.1038/s41598-020-61481-6 (PMC7076017; doi:10.1038/s41598-020-61481-6)
Supplement: Supplementary file 1 — Supplementary information. [file 41598_2020_61481_MOESM1_ESM.pdf]

Supplementary Information

Observation of spin-polarized Anderson state around charge neutral point in graphene with Fe clusters

Jungmin Park[1,2], Inseon Oh[1], Mi-Jin Jin[1], Junhyeon Jo[1], Daeseong Choe[1], Hyung Duk Yun[1], Suk Woo Lee[1], Zonghoon Lee[1], Soon-Yong Kwon[1], Hosub Jin[3], Suk Bum Chung [4] and Jung-Woo Yoo[1*]

[1]School of Materials Science and Engineering-Low Dimensional Carbon Materials Center, Ulsan National Institute of Science and Technology, Ulsan, 44919, Republic of Korea

[2]Center for Scientific Instrumentation, Division of Scientific Instrumentation & Management, Korea Basic Science Institute, Daejeon, 34133 Korea.

[3]Department of Physics, Ulsan National Institute of Science and Technology, Ulsan, 44919, Korea

[4]Department of Physics, University of Seoul, Seoul, 02504, Korea

*E-mail: jwyoo@unist.ac.kr

S1. Raman shift

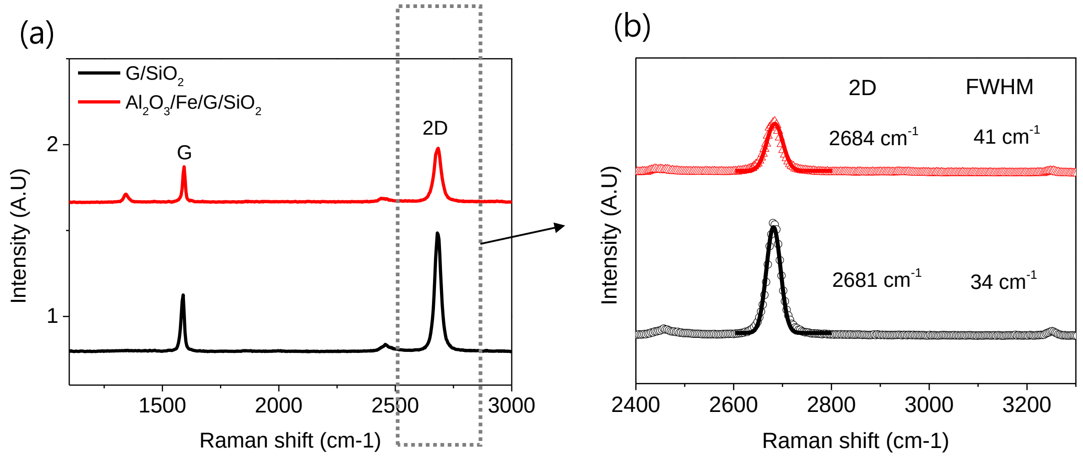

Figure S1. (a) Raman spectra of graphene and graphene with Fe clusters on SiO_2. The Al_2O_3 was deposited to protect Fe from oxidizing. (b) Enlarged view of Raman spectra for the region of 2D peak. Spectra was recorded at room temperature with wave length 532 nm. The solid lines in figure (b) are a fit to gaussian function. The 2D peak of graphene on SiO_2 was slightly shifted from 2681 cm^{-1} to 2684 cm^{-1}, and the full-width-at-half-maxima (FWHM) of 2D peak has increased about 12% after covering with Fe-adatoms and Al_2O_3 capping layer. Because double resonance process (2D band) involving two phonons is sensitive to the change in band structure induced by strain[1,2], the shift and broadening of 2D peak reflects Fe adatoms and Al_2O_3 capping layer induced significant strain in underlying graphene[3].

S2. Kondo behavior of graphene with Fe clusters.

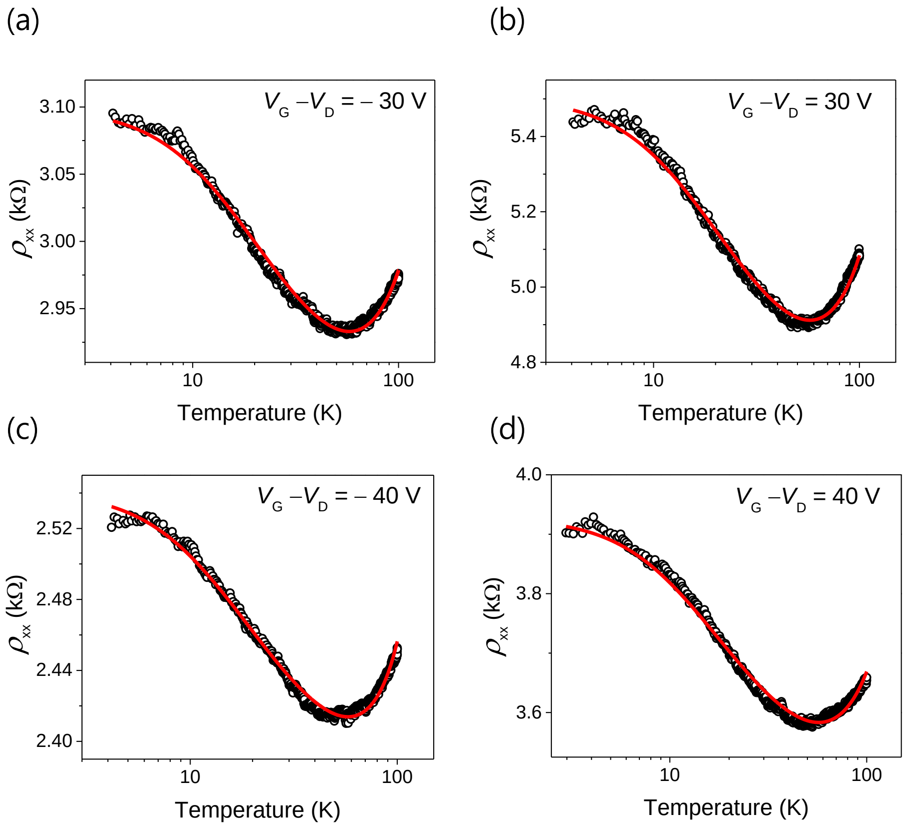

Figure S2. (a) - (d) a Kondo behavior of Fe-adatoms graphene for high carrier density, $V_G - V_D$ = − 30, 30, −40 and 40 V, respectively. The rad line is a fit using by simple Kondo model,

$$R_{\mathrm{kon}}(T) = R_0 + qT^2 + pT^5 + R_K(\frac{T}{T_K}) \quad (1)$$

where R_0 represents the resistance from sample disorder. T^2 and T^5 terms are the electron-electron and electro-phonon interaction, respectively. T_K indicates Kondo temperature. For the fitting of simple Kondo model to the data, we used the empirical form for universal resistivity function[4],

$$R_K\left(\frac{T}{T_K}\right) = R_K(T = 0)\left(\frac{T_K'^2}{T^2 - T_K'^2}\right)^S \quad (2)$$

where $T_K' = T_K/(2^{1/s}-1)^{1/s}$. The expected value of s in the Kondo regime depends on magnetic impurity and $s = 0.22\pm0.01$ for σ=1/2 was obtained by renormalization group[4]. Here, we fixed s = 0.21 for fitting. For $V_G - V_D$ = − 30 V , + 30 V , − 40 V , and +40V, a numerical

fit using equation (1) to measured R(T) curve yielded R_0 = 2644, 3937, 2238, and 3127 Ω , q = 0.013, 0.049, 0.0081, and 0.023 Ω/K^2, $p = 1 \times 10^{-11}$, 9×10^{-10}, 2×10^{-9}, and 5.8×10^{-10} Ω/K^5 ,respectively. The Kondo temperature for V_G–V_D = -30 V , $+30$ V , -40 V , and $+40$ V was 62, 63, 55, and 50 K, respectively. This variation of the Kondo temperature depending on the electron density corresponds to the conventional Kondo model[5], which follows $T_K \sim e^{-n}$.

S3. Al_2O_3 graphene device.

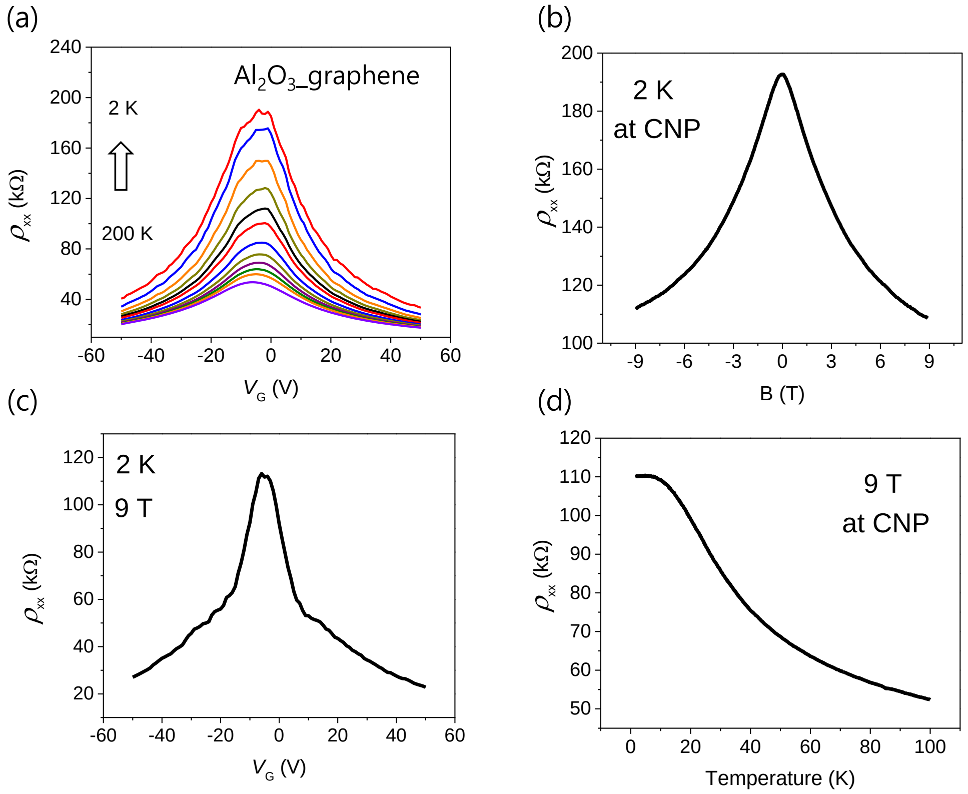

Figure S3. To compare influence of Fe adatoms on graphene, we investigate the charge transport properties of graphene on SiO_2 capped by Al_2O_3 layer without inclusion of ultrathin Fe. We have note that evaporation of Al_2O_3 on graphene induces random defects and disorder, as reported earlier[6,7]. (a) Gate dependent resistivity (ρ_{xx}) in control device measured at various temperature. Unlike Fe-adatoms graphene system, insulator behavior was observed over the wide range of carrier concentration. (b) Magnetoresistance measured at CNP and 2 K. Results displays strong negative magnetoresistance. (c) ρ_{xx} vs V_G measured at 2 K and 9 T, displaying absence of quantum Hall effect. (d) ρ_{xx} as a function of temperature measured with applied magnetic field of 9 T. ρ_{xx} increases with decreasing temperature exhibiting insulating behavior due to band gap opening.

S4. Longitudinal conductance *G* under high magnetic field.

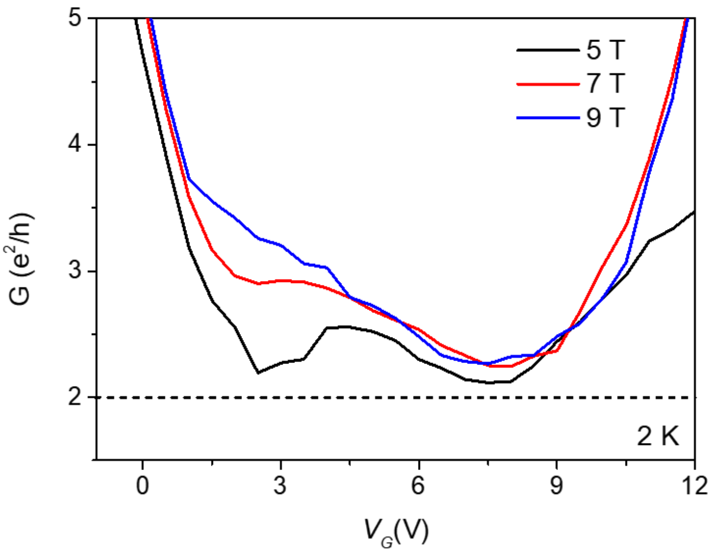

Figure S4. Longitudinal conductance *G* near CNP under magnetic field 5, 7, and 9 T. Results clearly display shift of CNP with increasing magnetic field and base conductance of $2(e^2/h)$ at CNP.

Reference

1 Ni, Z. H. *et al.* Uniaxial Strain on Graphene: Raman Spectroscopy Study and Band-Gap Opening. *ACS Nano* **2**, 2301-2305 (2008).

2 Huang, M., Yan, H., Heinz, T. F. & Hone, J. Probing Strain-Induced Electronic Structure Change in Graphene by Raman Spectroscopy. *Nano Lett.* **10**, 4074-4079 (2010).

3 He, R. *et al.* Large Physisorption Strain in Chemical Vapor Deposition of Graphene on Copper Substrates. *Nano Lett.* **12**, 2408-2413 (2012).

4 Goldhaber-Gordon, D. *et al.* From the Kondo Regime to the Mixed-Valence Regime in a Single-Electron Transistor. *Phys. Rev. Lett.* **81**, 5225-5228 (1998).

5 C, H. A. The Kondo Problem to Heavy Fermions. *Cambridge: Cambridge University Press* (1997).

6 Tang, X. *et al.* Damage evaluation in graphene underlying atomic layer deposition dielectrics. *Sci. Rep.* **5**, 13523 (2015).

7 Zheng, L. *et al.* Improvement of Al_2O_3 Films on Graphene Grown by Atomic Layer Deposition with Pre-H_2O Treatment. *ACS Appl. Mater. Interfaces* **6**, 7014-7019 (2014).
